# Design and use of a novel substrate for simple, rapid, and specific early detection of anthrax infection

**DOI:** 10.1371/journal.pone.0207084

**Published:** 2018-11-09

**Authors:** Kayana Suryadi, Nancy Shine

**Affiliations:** Research and Development, List Biological Laboratories, Inc., Campbell, California, United States of America; Stanford University, UNITED STATES

## Abstract

*Bacillus anthracis* is a major biological warfare threat. The inhalation form of infection can kill quickly. While antibiotic treatment is effective, if diagnosis is delayed, the rapidly produced toxin may already be present in lethal amounts. This report describes a fast, sensitive, specific and accurate method for detection of active infection by *Bacillus anthracis* in plasma. One of the virulence factors, anthrax lethal factor, is an endopeptidase present in blood early in the infection. However, the use of peptidic substrates to detect endopetidases is problematic in plasma due to the presence of other proteases and the likelihood of nonspecific cleavage of the substrate. The fluorescently labeled peptide substrate MAPKKide Plus designed in this study is not cleaved by plasma proteases and thus is specific for lethal factor. Three detection strategies are described. Two include enrichment by capture from plasma using lethal factor antibody-coated microtiter plates or similarly coated immuno-tubes. The captured lethal factor is exposed to the MAPKKide Plus, and the amount of cleavage is determined either by HPLC or microplate reader. Concentration of lethal factor using the antibody-coated plates aplnd HPLC allows for detection of less than 5 pg lethal factor/ml of neat plasma after 2 hours of incubation. Using antibody-coated immuno-tubes, 20 pg lethal factor/ml plasma can be detected in 5 hours by a simple end point read of fluorescence in a microplate reader. For a third strategy, the substrate is added directly to diluted plasma, and cleavage is monitored by the increase in fluorescence as a function of time. The limit of detection by this simple method is 25 ng lethal factor/ml of plasma in 15 minutes, 5 ng/ml after 45 minutes, and <1 ng lethal factor/ml of plasma after 5 hours.

## Introduction

*Bacillus anthracis* is the etiological agent of anthrax [1, 2]. The principal virulence factors are a γ-linked poly-D-glutamic acid (PGA) capsule and a three-component exotoxin composed of protective antigen (PA), lethal factor (LF) and edema factor (EF). These proteins act in binary combinations. The complex of PA, the cell binding component, with the LF enzyme is termed lethal toxin and can cause death. PA and the enzymatic EF together cause skin edema [3]. Secreted PA is cleaved by membrane peptidases [4] allowing the 63 kDa carboxy terminal fragment to oligomerize to a heptamer or larger [5]. This is an essential step in exposing the binding sites for EF and LF. The complex enters the cell through endocytosis [6]. PA mediates the transfer of LF and EF to the cytoplasm where these enzymes recognize and cleave their substrate targets.

There are three routes of human anthrax intoxication: cutaneous, inhalation, and gastrointestinal. About 95% of anthrax cases are cutaneous which is readily diagnosed and responds well to antimicrobial therapy [7]. With inhalational anthrax, however, as occurred in the 2001 intentional release of anthrax spores, early symptoms were similar to those for common illnesses [8]. The incubation period varies depending on the dose received. When symptoms are severe and a definitive diagnosis is possible, the levels of toxins can be dangerously high. Once intoxication occurs, anthrax bacteria can multiply rapidly in the blood and begin to secrete significant quantities of the toxins [9]. A quick, reliable test is needed to detect exposure early in the infection process. Standard microbiological tests to identify the bacteria involve lengthy procedures and may not be definitive. Confirmation of a diagnosis requires further analysis and may impede the rapid response needed for effective treatment. There are a number of markers of inhalation anthrax infection including PA, LF and PGA; however, it has been shown that LF is detectable earliest in the infection [10]. An overview of anthrax detection methods has been published recently [11]. Highly sensitive, activity-based assays to detect LF in serum and plasma have been reported using either matrix-assisted laser desorption/ionization time-of-flight mass spectrometry (MALDI-TOF-MS) or electrospray ionization MS/MS [12–15]. Levels as low as 10 pg LF/ml can be detected with a peptide substrate after a 2 hr incubation of the LF captured from the serum on antibody-coated beads [15]. One drawback to these procedures is that they require sophisticated instrumentation and a skilled operator.

The above methods take advantage of the fact that anthrax LF is an endoprotease and monitor the cleavage of a peptide substrate. LF cleaves the N-terminus of mitogen-activated protein kinase kinases (MAPKK). Specific peptide bonds hydrolyzed within various MAPKKs have been identified (e.g. MEK1, MEK2, MKK3, MKK4, MKK6, and MKK7) [16]. In addition, structural studies of LF complexed with peptide and inhibitor ligands have been reported [17]. A quenched fluorogenic (FRET) peptide substrate for LF, currently marketed as MAPKKide, was designed at List Biological Laboratories. FRET substrates are well suited for high throughput screening and identification of potential toxin inhibitors as therapeutic agents [18–22]. Measurement of proteolytic activity provides a potentially sensitive and direct means for detecting LF. Substantial signal amplification can be expected as a result of catalytic turnover. These FRET peptide substrates are based on the native sequences and retain a stretch of basic amino acids which have been shown to match with the strongly acidic region of LF [17]. As such, these sequences can be cleaved nonspecifically by other proteases in complex matrices such as plasma and serum, limiting their usefulness for detection of LF.

This work reports the design of fluorogenic peptide substrates which are resistant to nonspecific proteases and therefore specific for LF. Significantly, these newly designed substrates are highly sensitive to LF and may be used to detect early infections. Three sensitive methods for the detection of clinically relevant amounts of LF in plasma using one of these fluorogenic substrates, MAPKKide Plus [23], are described: one using HPLC and two using a microplate reader.

## Materials and methods

Anthrax lethal factor (Product #169L or Product #172L), MAPKKide Plus (Product #532) and the chicken IgY polyclonal anti-LF antibody (Product #769A) are products of List Biological Laboratories, Inc. The C8 Starwell Maxi Nunc-Immuno Module Plates (Cat #441653), the Nunc-Immuno Tubes, Maxisorp (Cat #444202) used for LF antibody coating and the dimethyl sulfoxide (DMSO) (Cat #TS-20684) are purchased from ThermoScientific. The 96-well black flat bottom non-binding plates used for the fluorescent plate assays are from Corning (Cat #3991). Bovine plasma (Cat #7310806) and ovine plasma (Cat #7319006) were purchased from Lampire Biological Laboratories. Milk (2% fat) came from a local market.

The peptide sequences evaluated as potential specific substrates for LF are shown in **Table 1**. All peptide substrates were synthesized using standard solid phase techniques. The peptides were shown to be >95% pure by reverse phase HPLC/UV. The expected molecular weights were confirmed by mass spectrometry. The peptide content was determined by nitrogen analysis. The N-terminal residue of each peptide substrate was acetylated.

**Table 1:** N-acyl fluorogenic substrates evaluated as potential LF substrates.

Substrate LBL#	P7	P6	P5	P4	P3	P2	P1	Attached Fluorophore	Purpose
**10079**	Arg	Arg	Lys	Lys	Val	Tyr	Pro	7-amido-4-methylcoumarin	To confirm the D-amino acids are essential for LF specificity
**10081**	Arg	D-Arg	D-Lys	D-Lys	Val	Tyr	Pro	7-amido-4-methylcoumarin	To determine the importance of chirality of Arg at P7
**10097**	D-Arg	D-Arg	D-Lys	D-Lys	Val	Tyr	Pro	7-amido-4-methylcoumarin	
**10100**	—	—	D-Lys	D-Lys	Val	Tyr	Pro	7-amido-4-methylcoumarin	To estimate the minimal length peptide necessary for efficient cleavage
**10108**	Arg	D-Arg	D-Lys	D-Lys	Val	Tyr	Pro	7-amido-4-trifluoro-methylcoumarin	To evaluate the effect of changing the released fluorophore
**10095**	(Arg	D-Arg	D-Lys	D-Lys	Val	Tyr	Pro)_2_	rhodamine	

### Sample preparation

#### Fluorogenic substrates

All stock solutions of the fluorogenic substrates were made in DMSO based on the peptide content and diluted in the appropriate optimized buffers described below. For the initial screen, all stock solutions for the substrates shown in **Table 1** were 5 mM in DMSO except for LBL 10095 which was 1.6 mM in 70% DMSO. The substrates were subsequently diluted in appropriate optimized assay buffers: LBL 10079, 10081, 10097 and 10100 were diluted in 20 mM HEPES, pH 8.0 containing 0.1% Tween-20; substrate LBL 10095 was diluted in 20 mM HEPES, pH 8.0 containing 0.1% Tween-20 and 20 μM ZnCl_2_; substrate LBL 10108 was diluted in 20 mM HEPES, pH 8.2 containing 0.1% Tween-20.

Based on the results obtained in the initial screens, and due to its superior performance, LBL 10081, currently marketed as MAPKKide Plus, was used in all subsequent assays. Stock solutions were in 1.25 or 2.5 mM DMSO for the HPLC and the rapid microplate assays, followed by dilution in the reaction buffer 20 mM HEPES, pH 8.0 containing 0.1% Tween-20. The final concentration was 1.25 μM or 2.5 μM MAPKKide Plus in the reaction mixture. For the assay using the antibody-coated Nunc-Immuno Maxisorp Tubes, the MAPKKide Plus final concentration in the reaction was 10 μM.

#### Anthrax lethal factor in plasma

For the rapid microplate assay, given amounts of LF were spiked into neat bovine plasma and then diluted 1:10 or 1:5 in the reaction buffer. For the assays using the antibody-coated C8 Starwell plates or the immuno- tubes, the LF was added to neat bovine plasma and not diluted.

#### Anthrax lethal factor chicken antibodies

For the assays using the antibody-coated C8 Starwell plates, the chicken affinity purified polyclonal IgY antibody to anthrax lethal factor (List Prod # 769A) was diluted to 10 μg/ml in 0.05M sodium carbonate-bicarbonate pH 9.6 buffer and each well was coated with 150 μl of the 10 μg/ml solution for a final coating of 1.5 μg IgY/well. For assays using the immuno-tubes, each tube was coated with 1 mL of the same 10 μg/ml solution of anti-LF antibody (List Prod # 769A). Both the plates and the immuno-tubes were incubated with the IgY overnight at 2–8°C prior to use in the assay.

### LF activity assays

#### Microplate assay methods

These assays were performed using a SPECTRAmax GEMINI XS fluorescence microplate reader (Molecular Devices). The cleavage reaction was initiated by addition of the substrate.

For the initial screen of the six substrates shown in **Table 1**, the substrate concentration (2.5 μM) was optimized to minimize background while maintaining measurable cleavage. Solutions of LF ranging from 0.5 to 2 ng/ml in 1:10 diluted bovine plasma or assay buffer were prepared. For all experiments the fluorescence was monitored at 37°C hourly for 5 or 6 hours followed by an additional 18 to 18.5 hr overnight incubation at ambient temperature. For the 7-amido-4-methyl-coumarin (AMC) containing substrates, the excitation wavelength was 368 nm and emission was 452 nm with a cutoff filter at 435 nm. For the substrate containing 7-amido-4-trifluoromethyl-coumarin (LBL 10108) the excitation wavelength was 372 nm and emission was 489 nm with a cutoff filter at 420 nm. For the rhodamine-containing substrate, the excitation wavelength was 494 nm, the emission wavelength was 531 nm with a cutoff filter at 515 nm. All results are the average of duplicate wells for the samples and the average of twelve wells for the blanks.

A rapid assay method was evaluated for two ranges of LF: 10 to 1000 pg LF/ml 1:10 diluted bovine plasma and 5 to 250 ng/ml 1:5 diluted bovine plasma. Dilution of the bovine plasma was necessary in order to minimize background.

For the range 10 and 1000 pg/ml 1:10 diluted plasma, 1.25 μM MAPKKide Plus was added directly to the diluted bovine plasma and the time-dependent increase in fluorescence was monitored at 37°C hourly for 5 hours followed by an additional 19 hour overnight incubation at ambient temperature. The excitation wavelength was set to 368 nm and emission to 452 nm with a cutoff filter at 435 nm. The samples were run in replicates of four. For the blank samples containing no LF there were 3 sets of quadruplicates and the standard deviation was calculated from these three sets. At each time point, the plate was read 5 times to increase the precision of the fluorescence readings. The standard curve was analyzed using a linear regression fit forcing the intercept through the mean value of the blanks. The limit of detection was calculated from the normal distribution of the blank samples (mean + 3 stdev; n = 3 sets of quadruplicates) and calculated as pg LF/ml plasma using the standard curve.

Subsequently, 6 data sets were evaluated, 2 data sets per day for 3 consecutive days. The data is presented as the average of these 6 data sets, each with 4 replicate samples and 12 replicate blanks. At each time point, the plate was read 3 times to increase the precision of the fluorescence readings.

For the method detecting higher levels of LF (5 to 250 ng/ml 1:5 diluted bovine plasma), 10 μM MAPKKide Plus was added directly to the diluted bovine plasma, and the assays were run at 37°C using the kinetic mode of the plate reader with readings at 1 or 3 minute intervals. The samples were run in triplicate with 9 replicate blanks. The limit of detection was calculated from the normal distribution of the blank samples (mean + 3 stdev; n = 9).

A microplate assay method using antibody capture was also developed. Nunc-Immuno Maxisorp Tubes were incubated with anti-LF IgY overnight at 2–8°C and then washed 5 times with 1.5 mL each of phosphate-buffered saline (PBS) containing 0.05% TWEEN-20 (PBST). The anti-LF coated tubes were exposed to 1 mL of a series of LF concentrations ranging from 20 to 2000 pg/ml in neat bovine plasma. The tubes were incubated at 22°C for 1 hour, then washed 3 times with 1.5 ml PBST, and 1.0 ml of 10 μM MAPKKide Plus was added. The reaction was allowed to proceed for 3, 5 and 22 hours at 37°C. At each time point, 250 μl of the reaction mixture was removed and placed in a 96-well plate. The excitation wavelength was 368 nm and emission was 452 nm with a cutoff filter at 435 nm. Any excitation wavelength between 360 to 368 nm can be used.

The experiment included triplicate tubes for each sample and nine blank tubes. The standard curve was analyzed using a linear regression fit forcing the intercept through the mean of the blanks. For the blanks there were three sets of triplicates and the standard deviation was calculated from these sets of triplicates. The limit of detection was calculated from the normal distribution of the blank samples (mean + 3 stdev; n = 3 triplicates) calculated as pg LF/ml neat plasma.

#### HPLC assay method

After the C8 Starwell plates were incubated with the anti-LF IgY overnight at 2–8°C, they were washed three times with 0.1M Glycine-HCl, pH 2.5. This wash was included to remove residual LF retained with the antibody after the affinity purification and helped to minimize background fluorescence. After an additional 6 washes with PBST, the anti-LF coated wells were exposed to 300 μl of a series of LF concentrations: 2.5 to 20 pg/ml neat bovine plasma, ovine plasma or milk (2% fat). The plates were incubated at 22°C for 2 hours, then washed 6 times with PBST and 250 μl of 1.25 μM LBL 10081 (MAPKKide Plus) was added. The reaction was allowed to proceed for 2, 3.5 and 5 hours at 37°C and overnight at ambient temperature (RT). At each time point 200 μl of the reaction mixture was removed from replicate wells and placed in HPLC sample vials.

HPLC was performed using a Varian ProStar HPLC system (Agilent) with a Zorbax Eclipse Plus C18 reverse phase column 4.6 x 150 mm (Agilent) and a guard column containing the same resin. Solvent A was 0.1% TFA in water and solvent B was 0.1% TFA in acetonitrile. The 16 minute HPLC method was as follows: 25% B for 0.75 minutes; 25 to 45% B in 4.75 minutes; 45 to 100% B in 0.75 minutes; 100% B for 3.75 minutes; 100 to 25% B in 0.67 minutes and 5.34 minute equilibration with 25% B. The injection volume was 20 μl. The column effluent was monitored using an Hitachi fluorescence detector with excitation of 350 nm and emission of 450 nm to detect the free fluorophore, 7-amino-4-methylcoumarin, at the retention time of 4.8 minutes.

The samples were run in duplicate with 6 blank samples. The standard curve was analyzed using a linear regression fit forcing the intercept through the mean of the blanks. The limit of detection was calculated from the normal distribution of the blank samples (mean + 3 stdev; n = 6), calculated as pg LF/ml neat plasma or milk (2% fat), from the standard curve generated at each incubation time.

## Results

### Lethal factor substrate design

The substrate sequences were based on a 15-amino acid fluorescence resonance energy transfer (FRET) peptide which was shown by Turk et al. [17] to contain an optimal cleavage motif. The sequence, 7-methoxy-coumarin-4-acetyl (Mca)-Arg-Arg-Lys-Lys-Val-Tyr-Pro-Try-Pro-Met-Glu -2,4-dinitrophenol (DNP) -Thr-Ile-Ala, is cleaved by LF after the first proline. This sequence contains a number of potential protease cleavage sites suggesting that non-specific cleavage in plasma would be problematic.

One objective of the current study was to determine the minimal length of substrate efficiently and specifically cleaved by LF. In a previous publication Tonello et al. [24] had shown that LF would allow the substitution of a fluorophore moiety, 7-amido-4-methylcoumarin (AMC), at the site of cleavage. This would decrease the length of the 15-amino acid substrate to seven amino acids. Subsequently, six peptides with 5 and 7 amino acids were designed and synthesized as potential substrates for LF (**Table 1**). All of the peptides are designed so that cleavage releases a fluorophore, either 7-amino-4 methyl-coumarin, 7-amino-4-trifluoromethyl-coumarin or rhodamine, resulting in a measurable increase in fluorescence which should be proportional to the amount of cleavage. LBL 10100 was used to elucidate the minimum length necessary for efficient cleavage of the substrates.

The specificity of these substrates was enhanced by taking advantage of the chiral nature of the amino acids and the fact that plasma proteases process only L-amino acids [25]. In order to maximize specificity, D-amino acids were substituted in some or all of the positions P7-P4 (**Table 1**). The peptide LBL 10079, which contains the native L-amino acids, was evaluated to demonstrate that D-amino acids are necessary to maximize the specificity in plasma. Comparison of the efficiency of cleavage of LBL 10079 with substrates containing D-amino acids was performed to evaluate the effect that the D-amino acids have on the rate of cleavage by LF. In LBL 10081, the arginine at position P6 and the two lysines in position P5 and P4 are substituted with the equivalent D-amino acid. The N-terminal arginine at P7 was left as the L-amino acid since most trypsin-like proteases do not cleave at the N-terminus. Other aminopeptidases can cleave this N-terminal residue; however, this would not cause a false positive since fluorescence is only increased if the fluorophore at the C-terminus is released. Cleavage of the substrate at other positions would decrease the length and could reduce the concentration of cleavable substrate sufficiently to affect the detection of LF. The substrate LBL 10097 was synthesized to determine if substitution of D-amino acids for all four basic residues would have an effect on the cleavage by LF.

Based on the results obtained for the first four sequences listed in **Table 1**, LBL 10095 was synthesized containing the same sequence as LBL 10081 but with a rhodamine fluorophore. Rhodamine is conjugated with two peptide substrates per fluorophore [26]. Cleavage of both substrates was expected to give a maximum increase in fluorescence of all the peptides evaluated. The peptide LBL 10108 containing the fluorophore 7-amido-4-trifluoromethyl-coumarin was also evaluated in order to compare fluorophores.

### Initial screen of six potential lethal factor substrates using a microplate assay

#### Cleavage of fluorescent peptides by LF in assay buffer

**Fig 1:**
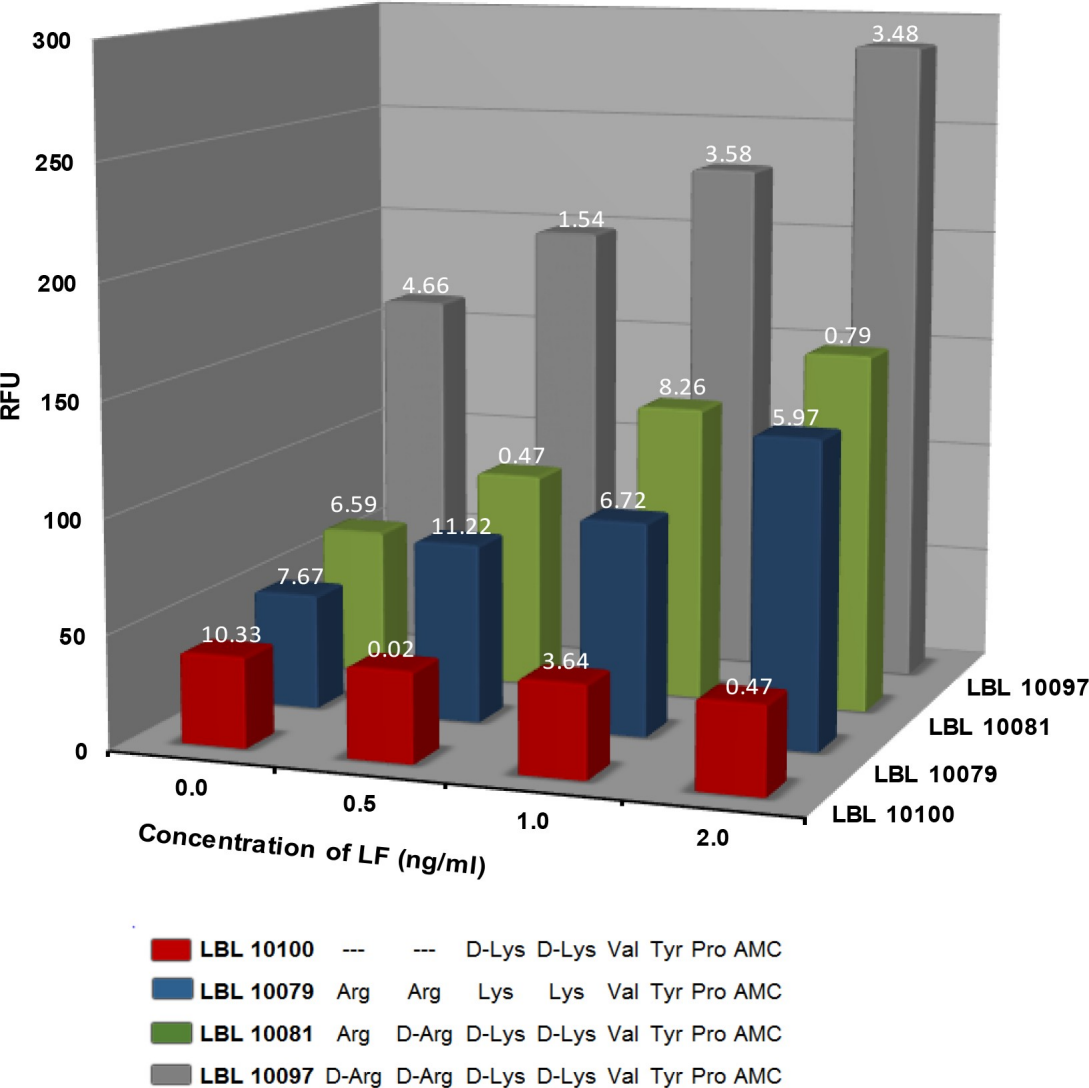
Cleavage of substrates in assay buffer. Plot of the increase in fluorescence (RFU) after 5 hours at 37°C as a function of LF concentration (ng/ml) for LBL 10100 (red), LBL10079 (blue), LBL 10081 (green) and LBL 10097(gray). LF samples were run in duplicate. The % difference of the duplicates is shown above each bar. The value shown for blanks (n = 6 duplicates) is the average of the % differences. These results were obtained using the microplate assay.

Cleavage of the four 7-amido-4-methylcoumarin-labeled substrates (2.5 μM) by 0.0, 0.5, 1, and 2 ng LF/ml assay buffer is shown in the bar graph of **Fig 1**. In assay buffer all the substrates, except LBL 10100, show fluorescence proportional to the concentration of LF.

The results of this study indicate that the minimum length demonstrated for efficient cleavage by LF is 7 amino acids. Removal of 2 amino acids from the N-terminus, as is the case for LBL 10100 (red bars), eliminates hydrolysis at the concentrations of LF used.

Despite the high background, cleavage of LBL 10097 with D-Arg at the N terminus (gray bars) is similar to that of LBL 10081 (MAPKKide Plus) with L-Arg at the N-terminus (green bars) indicating that the chirality of the N-terminal Arg does not significantly affect cleavage by LF.

The amount of hydrolysis observed for LBL 10079, which does not contain any D-amino acids (blue bars), is similar to that of LBL 10081(MAPKKide Plus) which contains three D-amino acids (green bars) indicating that the cleavage of these substrates by LF is not impaired by the substitution with D-amino acids.

The use of D-amino acids should improve the stability and specificity of LBL 10081(MAPKKide Plus) in plasma by inhibiting cleavage by other proteases.

#### Cleavage of fluorescent peptides by lethal factor in 1:10 diluted bovine plasma

**Fig 2:**
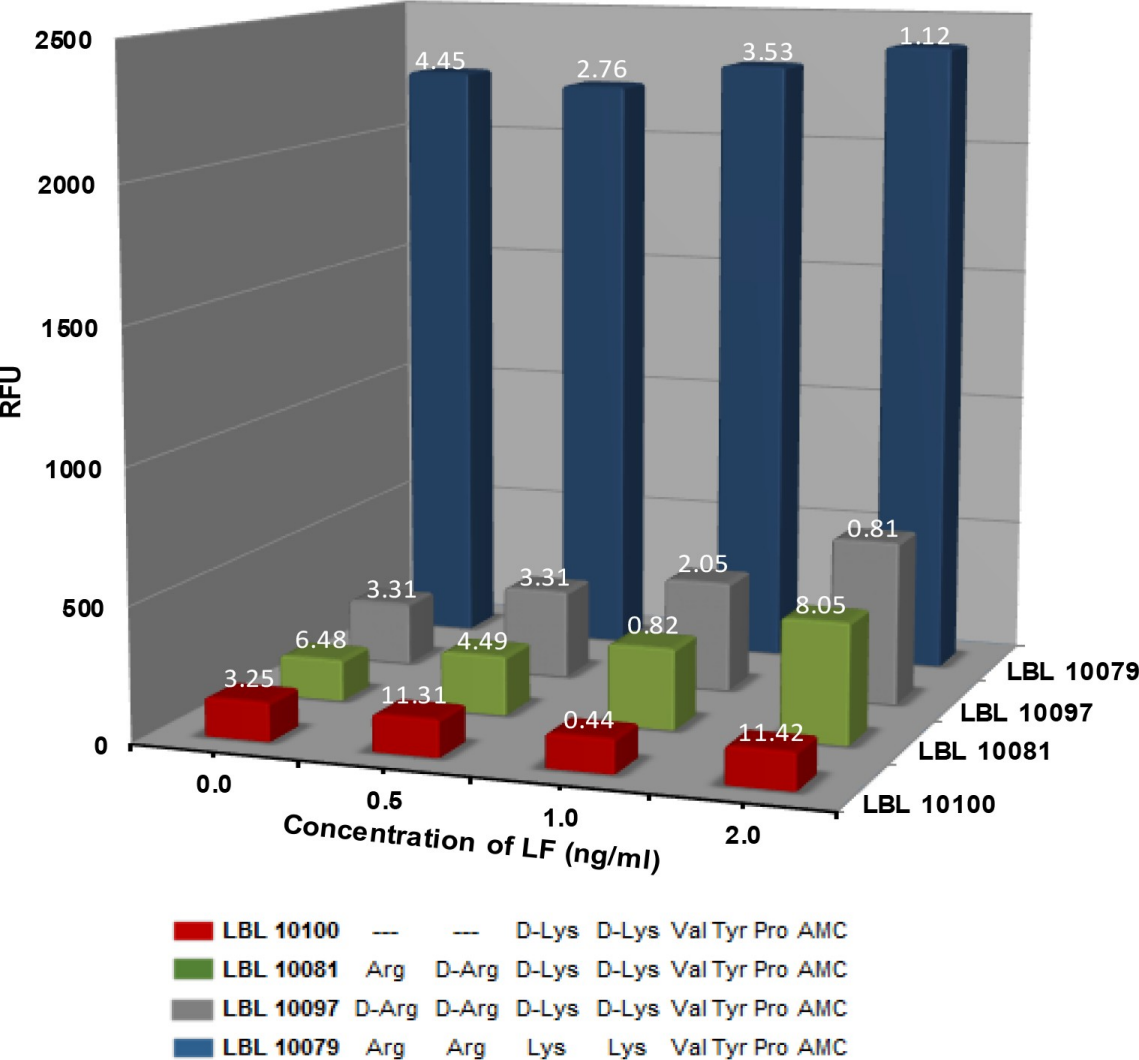
Cleavage of substrates in 1:10 diluted bovine plasma. Plot of the increase in fluorescence (RFU) after 5 hours at 37°C as a function of LF concentration (ng/ml) for LBL 10100 (red), LBL10081 (green), LBL 10097 (gray) and LBL 10079 (blue). LF samples were run in duplicate. The % difference of the duplicates is shown above each bar. The value shown for blanks (n = 6 duplicates) is the average of the % differences. These results were obtained using the microplate assay.

The bar graph in **Fig 2** presents data for cleavage of the four LF substrates containing the 7-amido-4-methylcoumarin fluorophore (2.5 μM) by LF in 1:10 diluted bovine plasma. Significant fluorescence is observed for LBL 10079 (blue bars) in plasma even in the absence of LF. This indicates that some plasma proteases cleave at the proline, releasing the fluorophore and giving rise to significant fluorescence. The cleavage does not correlate with the amount of LF in the sample. Non-specific cleavage at the Arg and Lys residues, which are L-amino acids in this substrate, would not explain this increased fluorescence since this does not generate the free fluorophore. However, plasma does contain proline proteases such as prolylcarboxypeptidase which cleave the last amino acid at the C-terminus if it is linked to a penultimate proline [27, 28]. Here the fluorophore is on the C-terminal residue linked to the proline. This activity might explain the observable non-specific cleavage of LBL 10079.

**Fig 3:**
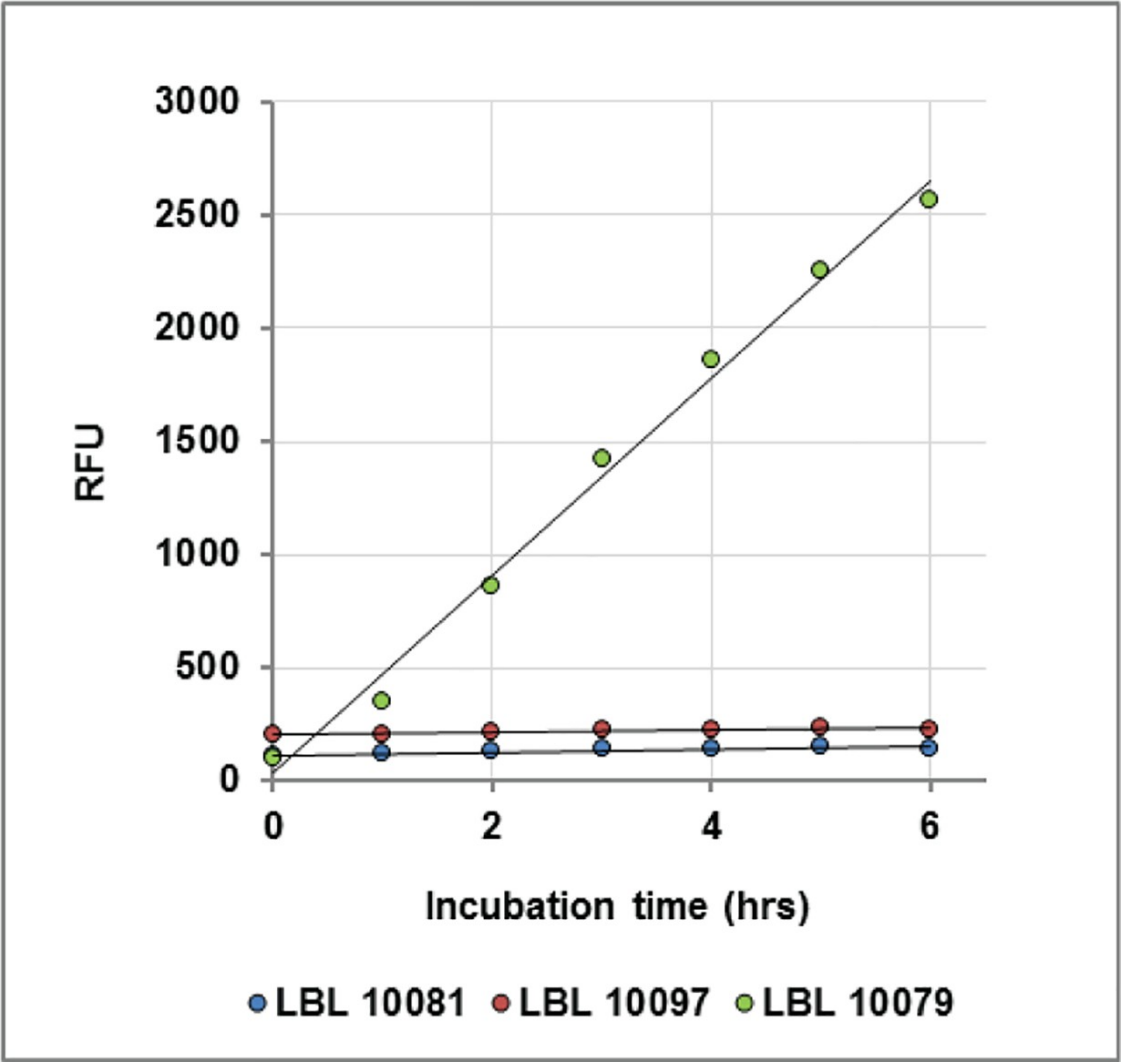
Substrate specificity in bovine plasma without LF. Fluorescence (RFU) as a function of time for LBL 10081 (blue), LBL 10097 (red) and LBL 10079 (green) in 1:10 bovine plasma at 37°C in the absence of LF.

The substitution of three or four of the amino acids with D-amino acids inhibits non-specific cleavage of LBL10081(MAPKKide Plus, green bars) and LBL 10097 (gray bars) by plasma proline proteases without affecting hydrolysis by LF. There is minimal increase in hydrolysis of these substrates in plasma without LF even after 5 hours incubation at 37°C and after an additional 18 hours at ambient temperature (see **Table 2, part A** and **Fig 3** below). This is also true for samples containing ten times the amount of substrate in more concentrated plasma, i.e. 25 μM substrate in 1:5 diluted plasma (see **Table 2, part B** below).

**Table 2:** Fluorescence observed for substrates in diluted bovine plasma.

**A: Fluorescence observed for 2.5** μM substrates in 1:10 diluted bovine plasma without LF*.										
**Time (hrs)**	**Temperature (°C)**	**LBL 10081** **RFU**	**Standard Deviation**	**%CV**	**LBL 10097** **RFU**	**Standard Deviation**	**%CV**	**LBL 10079** **RFU**	**Standard Deviation**	**%CV**
**0**	37	109	8.10	7.4	205	3.43	1.7	102	2.05	2.0
**1**	37	123	3.58	2.9	208	6.13	2.9	353	11.85	3.4
**2**	37	134	4.37	3.3	217	5.00	2.3	860	21.41	2.5
**3**	37	142	5.95	4.2	225	6.20	2.8	1420	38.26	2.7
**4**	37	144	5.27	3.7	228	6.66	2.9	1854	52.87	2.9
**5**	37	151	6.15	4.1	234	6.56	2.8	2251	62.75	2.8
**6**	37	147	6.13	4.2	227	5.81	2.6	2571	74.02	2.9
**+15**	ambient	163	7.79	4.8	243	6.03	2.5	4074	121.89	3.0
**+33**	ambient	169	7.37	4.4	236	5.93	2.5	3920	118.87	3.0
**B: Fluorescence observed for 25** μM substrates in 1:5 diluted bovine plasma without LF**.										
**0**	37	622	65	10.5	1307	118	9.0	558	45	8.1
**1**	37	572	16	2.8	1110	56	5.1	646	54	8.4
**2**	37	585	18	3.0	1111	48	4.4	1284	70	5.5
**3**	37	586	28	4.7	1092	79	7.2	1890	149	7.9
**4**	37	601	28	4.7	1115	51	4.6	2806	157	5.6
**5**	37	589	22	3.8	1121	56	5.0	3632	149	4.1
**6**	37	588	17	2.9	1112	62	5.6	4465	170	3.8
**+48**	ambient	760	51	6.7	1205	32	2.7	26027	949	3.6

*Statistics are based on 12 replicates; 3 reads per time point.

**Statistics are based on 6 replicates; excitation/emission wavelengths set to 360nm/460nm with no cutoff.

Based on the data above, two additional substrates with different fluorophores were synthesized to determine whether changing the fluorophore is advantageous. Both peptides contain the same amino acid sequence as LBL 10081(MAPKKide Plus). LBL 10108 contains a 7-amido-4-trifluoromethyl-coumarin group at the C-terminus. LBL 10095 consists of 2 peptides identical with LBL 10081 bound to rhodamine at their C-terminal ends. The fluorescence is proportional to the concentration of LF in 1:10 diluted plasma and neither peptide is cleaved in plasma in the absence of LF; however, there is no significant improvement in the sensitivity for either substrate when compared to LBL 10081 or LBL 10097.

The specificity observed for the D-amino acid containing substrates in plasma is further demonstrated in **Table 2, part A** and **Fig 3.** The change in fluorescence for 2.5 μM LBL 10081(MAPKKide Plus) and LBL10097 in the presence of 1:10 diluted plasma without LF is compared to LBL 10079. The results demonstrate that LBL 10081(MAPKKide Plus) and LBL 10097 are specific for LF and that LBL 10079 is non-specifically cleaved by plasma proteases. The cleavage of 25 μM of each of these substrates in 1:5 diluted bovine plasma was also evaluated (**Table 2, part B**). There is no evidence of nonspecific cleavage of LBL 10081 or LBL 10097 even at the increased concentrations of both plasma and substrate, confirming specificity.

### Optimized methods for detection of lethal factor activity in complex matrices using MAPKKide Plus

Based on the data presented above, all subsequent assays were performed using LBL 10081 (MAPKKide Plus).

#### HPLC method using antibody capture to concentrate the LF

**Fig 4:**
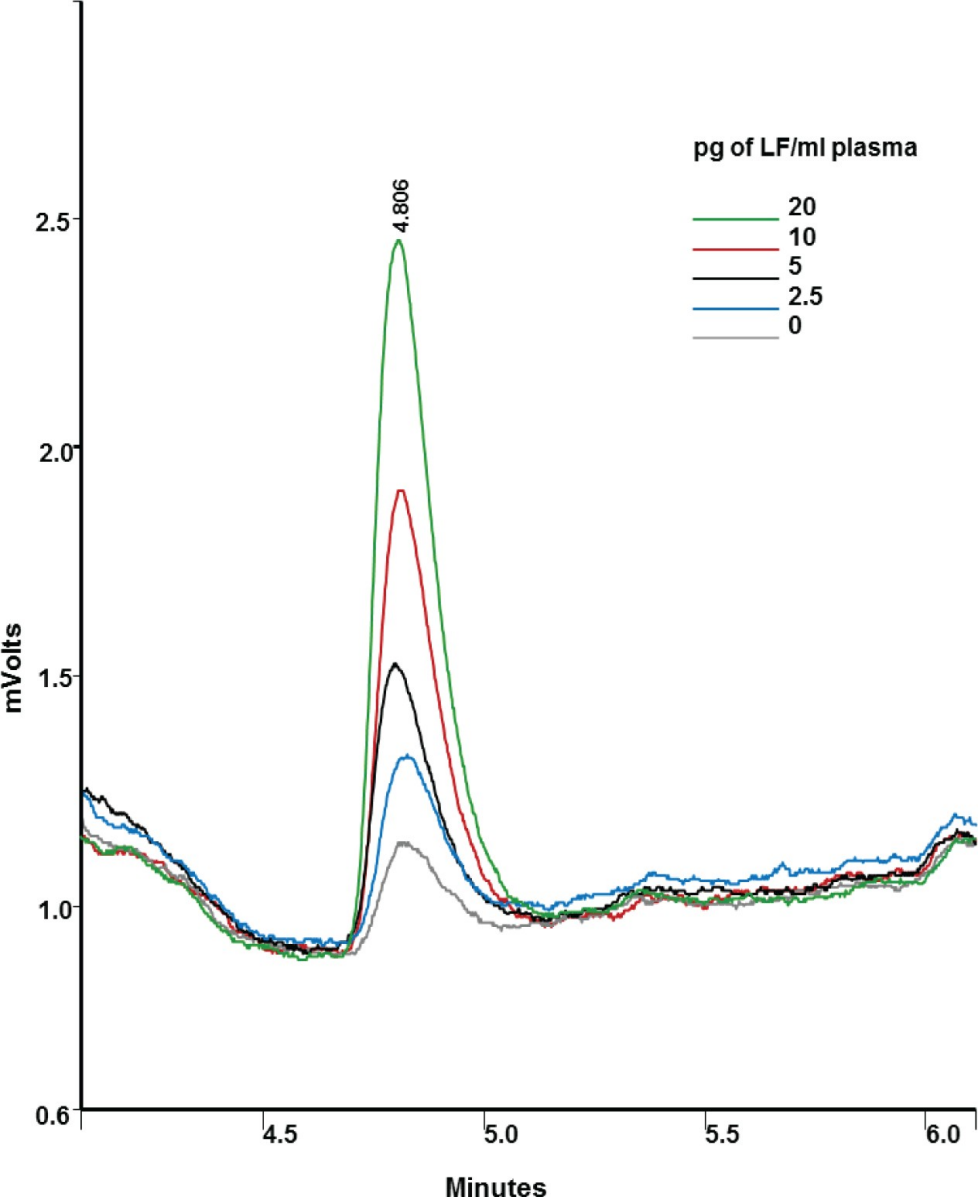
Detection of LF in neat bovine plasma using HPLC. Chromatograms from the digestion of MAPKKide Plus by 0 (gray), 2.5 (blue), 5 (black), 10 (red), and 20 (green) pg of LF/ml of neat bovine plasma after 2 hours at 37°C. The column effluent is monitored using an Hitachi fluorescence detector with excitation set to 350 nm and emission at 450 nm. The peak at 4.806 minutes is the cleaved 7-amino-4-methylcoumarin peak.

In order to optimize the detection of low levels of LF in complex matrices, the LF was enriched from neat plasma without dilution using the affinity purified anti-LF IgY coated on a 96-well microtiter plate. After addition of MAPKKide Plus, samples were monitored by HPLC with fluorescence detection after 2, 3.5 and 5 hrs of incubation at 37°C and overnight at ambient temperature. Representative chromatograms obtained for bovine plasma samples containing LF are shown in **Fig 4**.

The HPLC data obtained for neat bovine plasma, ovine plasma and milk (2% fat) is summarized in **Table 3**. Cleavage of MAPKKide Plus is similar in the three complex matrices.

**Table 3:** Detection of LF in neat bovine plasma, ovine plasma and milk (2% fat) using HPLC.

LF (pg/ml plasma or milk)	Average peak area*											
	2 hour digest			3.5 hour digest			5 hour digest			overnight digest (RT)		
	plasma		milk (2% fat)	plasma		milk (2% fat)	plasma		milk (2% fat)	plasma		milk (2% fat)
	bovine	ovine		bovine	ovine		bovine	ovine		bovine	ovine	
**0**	2380	3579	2781	3840	5306	3636	5033	7071	5501	7835	10678	8373
**2.5**	3696	5415	4441	6294	10455	6764	8788	11607	9001	14515	16268	15278
**5**	5459	6871	6212	8976	12210	9234	12218	14079	13241	21377	23760	23467
**10**	8875	9964	9694	14448	15536	15024	20487	22008	22257	34083	36140	38173
**20**	14311	14804	16150	25587	26007	27071	34502	36095	40754	62133	61944	66692

*Data is the average of duplicates for the samples and 6 replicates for the blanks.

**Fig 5:**
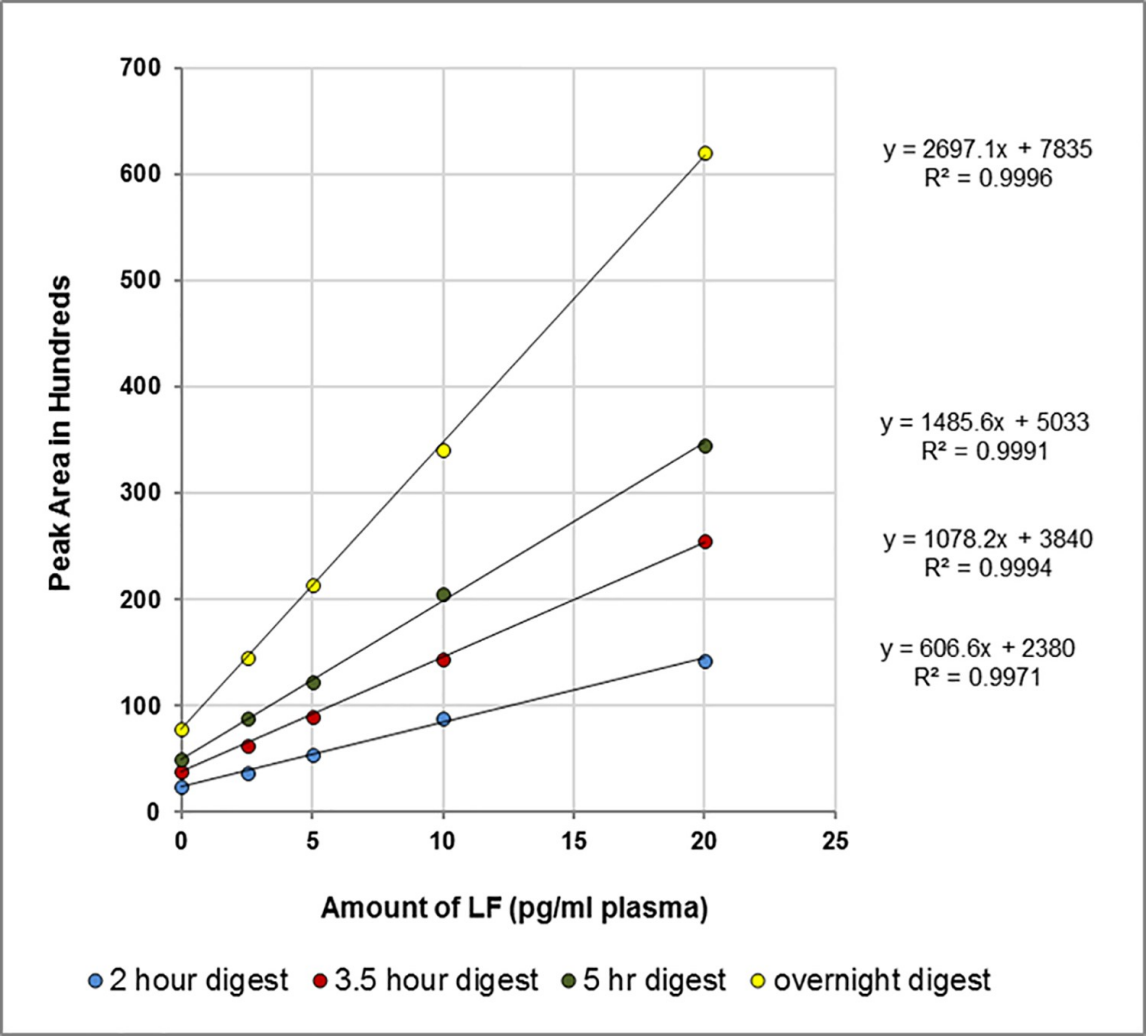
Plot of HPLC data. Plot of peak area versus concentration of LF (pg/ml plasma) for 2 hour (blue), 3.5 hour (red), 5 hour (green) and overnight (yellow) digestion of MAPKKide Plus.

Plots of peak areas as a function of LF concentration in bovine plasma are shown in **Fig 5**. Statistical analysis of the data from neat bovine plasma, ovine plasma, and milk (2% fat) are given in **Table 4**. Responses for all time points and matrices are linear.

**Table 4:** Slope, intercept, and correlation coefficients and LODs for bovine plasma, ovine plasma and milk (2% fat) at each incubation time.

Measurement	Incubation Time											
	2 hour			3.5 hour			5 hour			Overnight (RT)		
	Plasma		milk (2% fat)	Plasma		milk (2% fat)	Plasma		milk (2% fat)	Plasma		milk (2% fat)
	bovine	ovine		bovine	ovine		bovine	ovine		bovine	ovine	
**Slope (Peak** **Area/pg LF)**	607	582	674	1078	1061	1164	1486	1461	1732	2697	2559	2931
**Intercept (Peak area)**	2380	3579	2781	3840	5306	3636	5033	7071	5501	7835	10678	8373
**R**^**2**^	0.997	0.999	0.999	0.999	0.961	0.999	0.999	0.998	0.999	0.999	0.999	0.999
**Std deviation of the blanks**	785	337	979	817	576	175	680	318	861	469	743	738
**Average + 3x standard deviation**	4734	4589	5717	6290	7034	4161	7074	8024	8083	9242	12907	10587
**LOD (pg/ml plasma)**	3.88	1.73	4.36	2.27	1.63	0.45	1.37	0.65	1.49	0.52	0.87	0.76

The limit of detection for all three complex matrices is less than 5 pg LF/ml after 2 hours of incubation, less than 3 pg LF/ml in 3.5 hours and less than 2 pg LF/ml after 5 hours and overnight.

#### Kinetic analysis of the LF activity by HPLC

**Fig 6:**
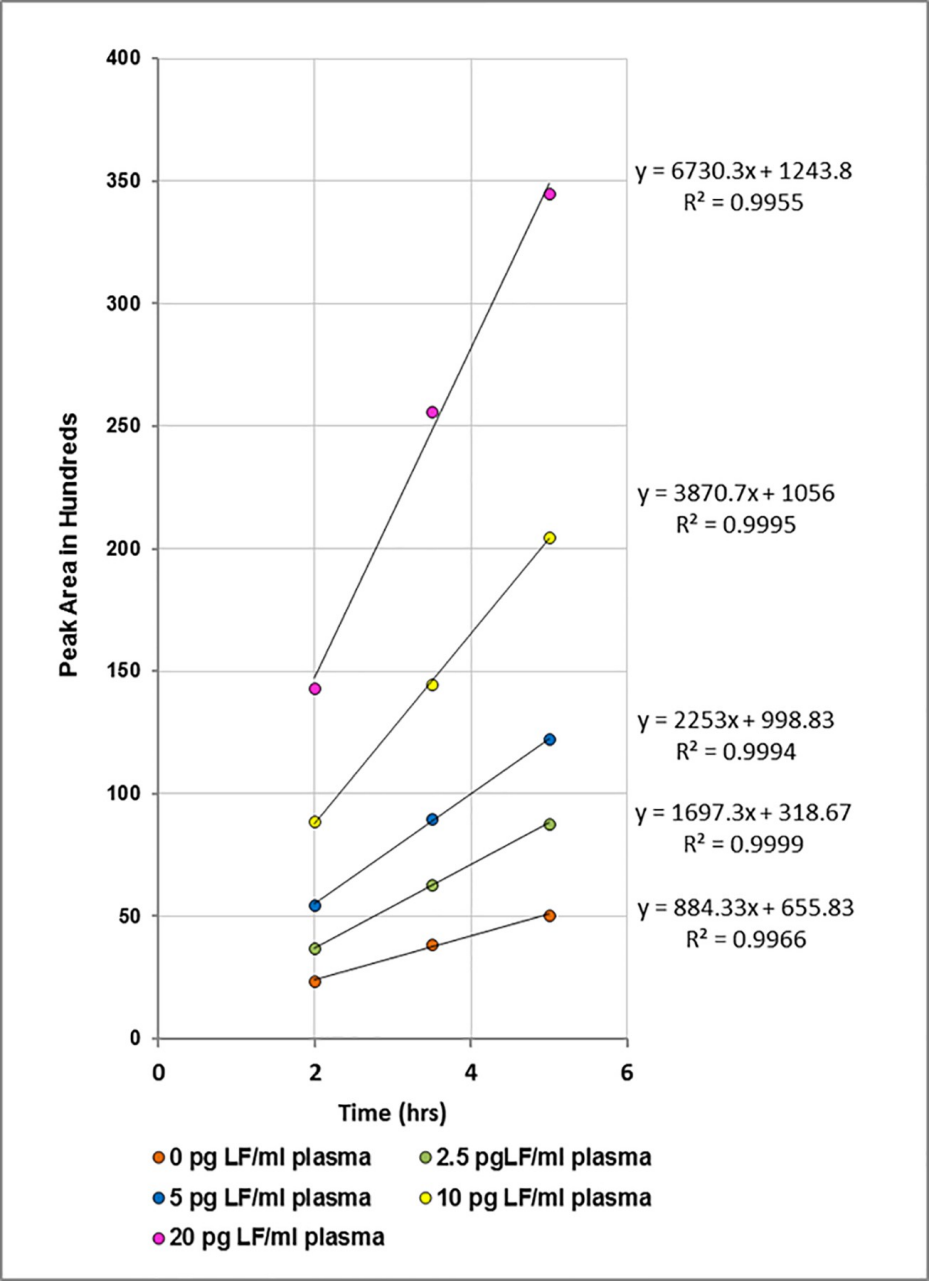
Kinetic analysis of HPLC data obtained for neat bovine plasma. Plot of peak area versus incubation time for 0 (orange), 2.5 (green), 5 (blue), 10 (yellow), and 20 (pink) pg LF/ml bovine plasma.

**Fig 7:**
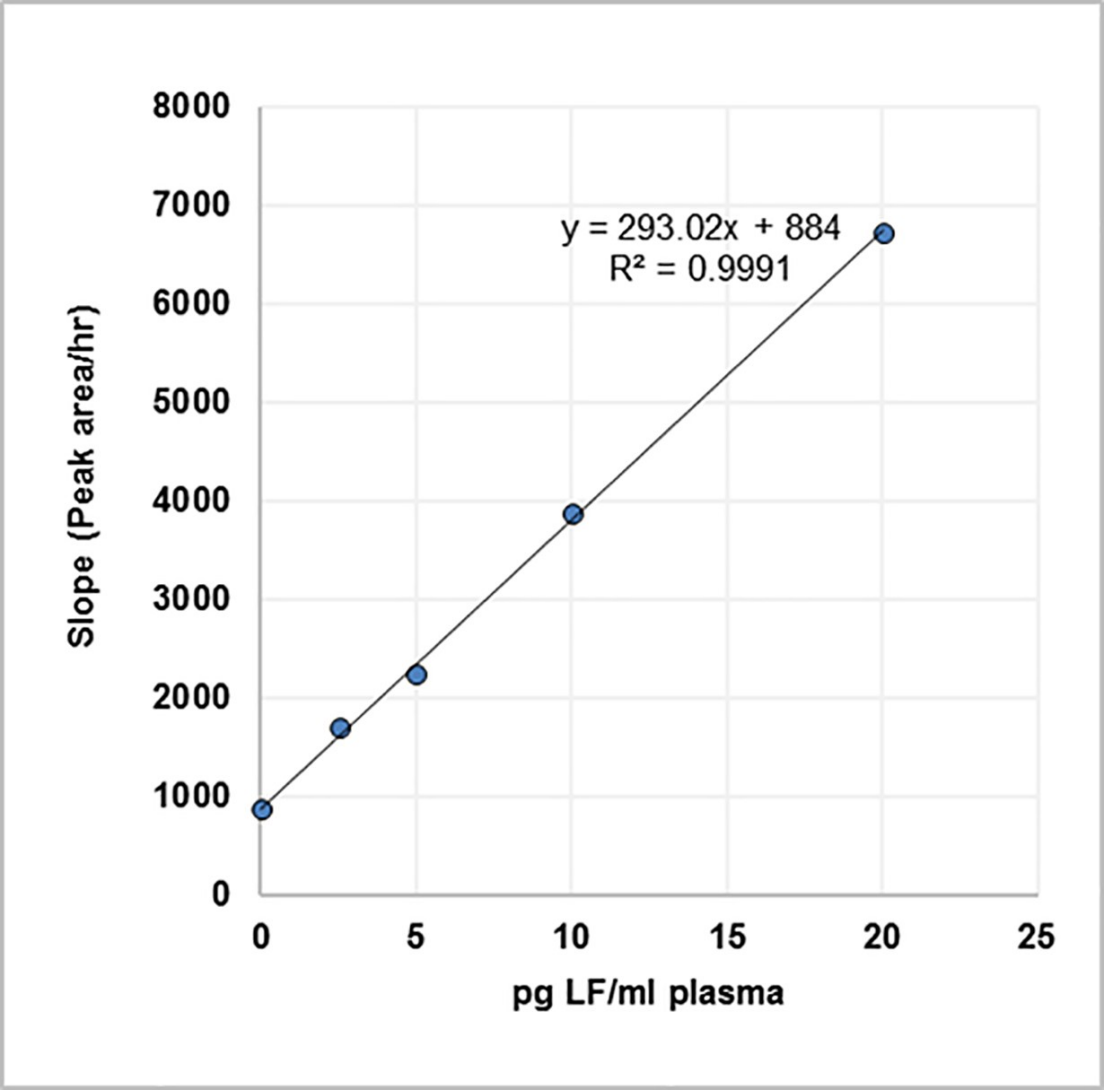
Rate of cleavage of MAPKKide Plus as a function of LF concentration. Plot of the slopes as a function of the concentration of LF (pg/ml bovine plasma).

The amount of LF in a sample can be determined from any single time point as shown above. However, such results in unknown samples might be subject to false positives due to uncertain background fluorescence. Alternately, or as confirmation of positive outcomes, the results can be obtained by monitoring the reaction rate, i.e. the increase in peak area as a function of time. Plots of peak area versus incubation time for the neat bovine plasma data is shown in **Fig 6**. Each concentration of LF yields a unique slope (rate) which is proportional to concentration (**Fig 7**). The limit of detection by this method is also around 5 pg LF/ml neat bovine plasma.

#### Rapid microplate assay methods

Two microplate assay strategies were evaluated. One involved adding the MAPKKide Plus directly to diluted bovine plasma and the other tested the use of antibody-coated immuno-tubes to capture the LF from neat bovine plasma prior to incubation with MAPKKide Plus.

**Fig 8:**
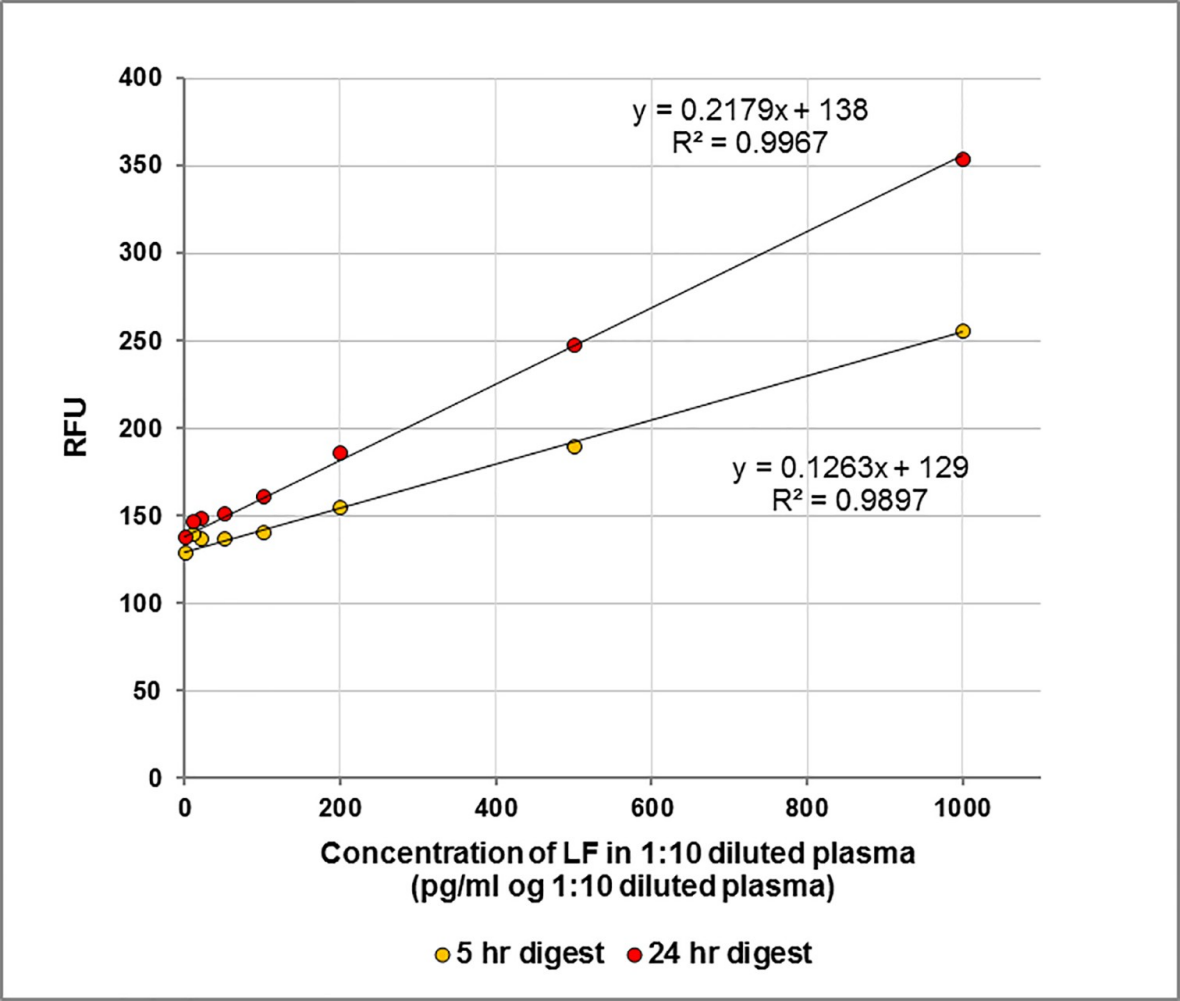
Cleavage of MAPKKide Plus monitored in the microplate assay. Cleavage at 5 hrs (yellow circles) and 24 hrs (red circles), as a function of LF concentration in 1:10 diluted bovine plasma.

In the simpler method the substrate is added directly to diluted bovine plasma in replicate microplate wells and the fluorescence monitored hourly. A plot of the cleavage of 1.25 μM MAPKKide Plus at 5 hrs (yellow circles) and 24 hrs (red circles) as a function of LF concentration is shown in **Fig 8**. The amount of cleaved peptide is linearly proportional to the amount of LF present in the diluted bovine plasma from 10 to1000 pg LF/ml of diluted bovine plasma. The data is shown in **Table 5**. The limit of detection after 5 hrs and 24 hrs digestion is approximately 2 ng LF/ml and 1 ng LF/ml neat bovine plasma, respectively.

**Table 5:** Digestion of MAPKKide Plus by different concentrations of LF in 1:10 diluted bovine plasma.

	LF (pg/ml) in 1:10 diluted plasma*								LOD	
RFU data	1000	500	200	100	50	20	10	0	pg LF/ml of 1:10 diluted plasma**	pg LF/ml neat plasma
**RFUs after 5 hrs digest**	256	190	155	141	137	137	140	129	32	320
**Standard Deviation**	11.37	6.38	6.21	11.13	4.36	5.72	5.48	1.33		
**%CV**	4.4	3.4	4.0	7.9	3.2	4.2	3.9	1.03		
** **										
**RFUs after 24 hrs digest**	354	248	186	161	152	149	147	138	38	380
**Standard Deviation**	21.64	8.38	6.67	11.25	5.52	6.28	4.17	1.60		
**%CV**	6.1	3.4	3.6	7.0	3.6	4.2	2.8	1.16		

* The samples were run in replicates of four. Twelve replicates were run for samples containing no LF. At each time point, the plate was read 5 times.

**The LOD was estimated from the normal distribution of the blank samples (mean + 3 stdev; n = 3 quadruplicates) and calculated as pg LF/ml plasma using the standard curve.

#### Kinetic analysis of the LF activity using the rapid microplate assay

The data above is for single endpoints after 5 and 24 hours digestion. As discussed previously, a kinetic treatment of the data can be used to confirm positive results and accommodate varying backgrounds.

**Fig 9:**
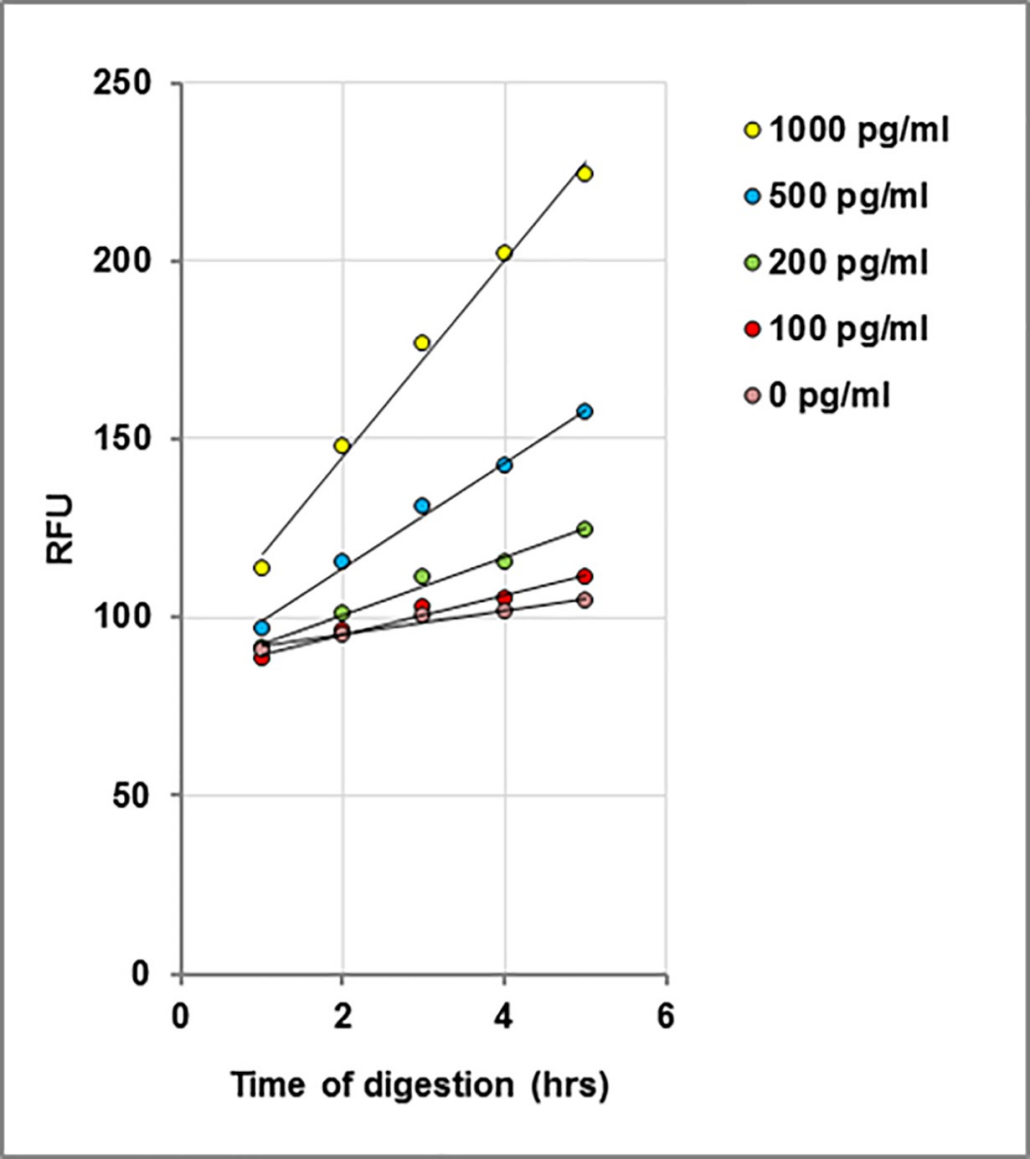
Kinetic analysis of microplate assay data. Cleavage of MAPKKide Plus by a series of concentrations of LF(pg/ml) in 1:10 diluted bovine plasma as a function of time.

**Fig 10:**
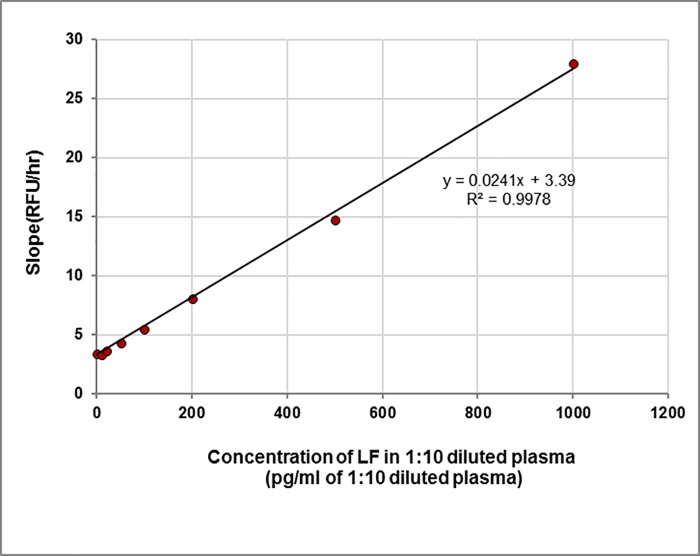
Rate of cleavage of MAPKKide Plus as a function of LF concentration. Slope in RFU/hour as a function of concentration of LF in 1:10 diluted bovine plasma.

The results from six data sets over three days were averaged and are presented in **Table 6**. Plots of cleavage as a function of time for individual LF concentrations are shown in **Fig 9**, each concentration yielding a unique slope. These slopes are then plotted as a function of concentration (**Fig 10**), which can be used to determine the amount of LF present in unknown samples. The limit of detection, calculated from the normal distribution of the slopes (mean + 3 stdev; n = 6), was 80 pg/ml of 1:10 diluted bovine plasma. This is equivalent to 800 pg LF/ml in neat bovine plasma.

**Table 6:** Digestion of MAPKKide Plus by different concentrations of LF in 1:10 diluted bovine plasma as a function of time.

Time (hours)	Substrate RFUs as a function of LF concentration (pg/ml 1:10 diluted plasma)*											
	1000		500		200		100		50		0	
	RFU	%CV	RFU	%CV	RFU	%CV	RFU	%CV	RFU	%CV	RFU	%CV
**1**	114	7.4	97	9.1	92	7.4	88	8.6	88	7.9	91	9.5
**2**	148	4.1	116	8.6	101	7.1	96	5.1	95	7.3	95	8.1
**3**	177	5.8	131	8.1	111	6.7	103	6.8	100	8.1	101	7.7
**4**	202	2.4	142	6.0	115	5.2	105	4.4	102	6.2	102	6.7
**5**	224	3.4	157	5.0	125	4.7	111	6.2	106	5.3	105	7.3
**Slope (RFU/hr)**	**27.48**	**5.3**	**14.72**	**3.6**	**8.06**	**5.7**	**5.46**	**9.9**	**4.30**	**13.7**	**3.39**	**16.2**

*RFU’s are the averages from 6 data sets, each with 4 replicates for each sample which contains LF and 12 blank replicates per data set. Three plate reads at each time point.

**Fig 11:**
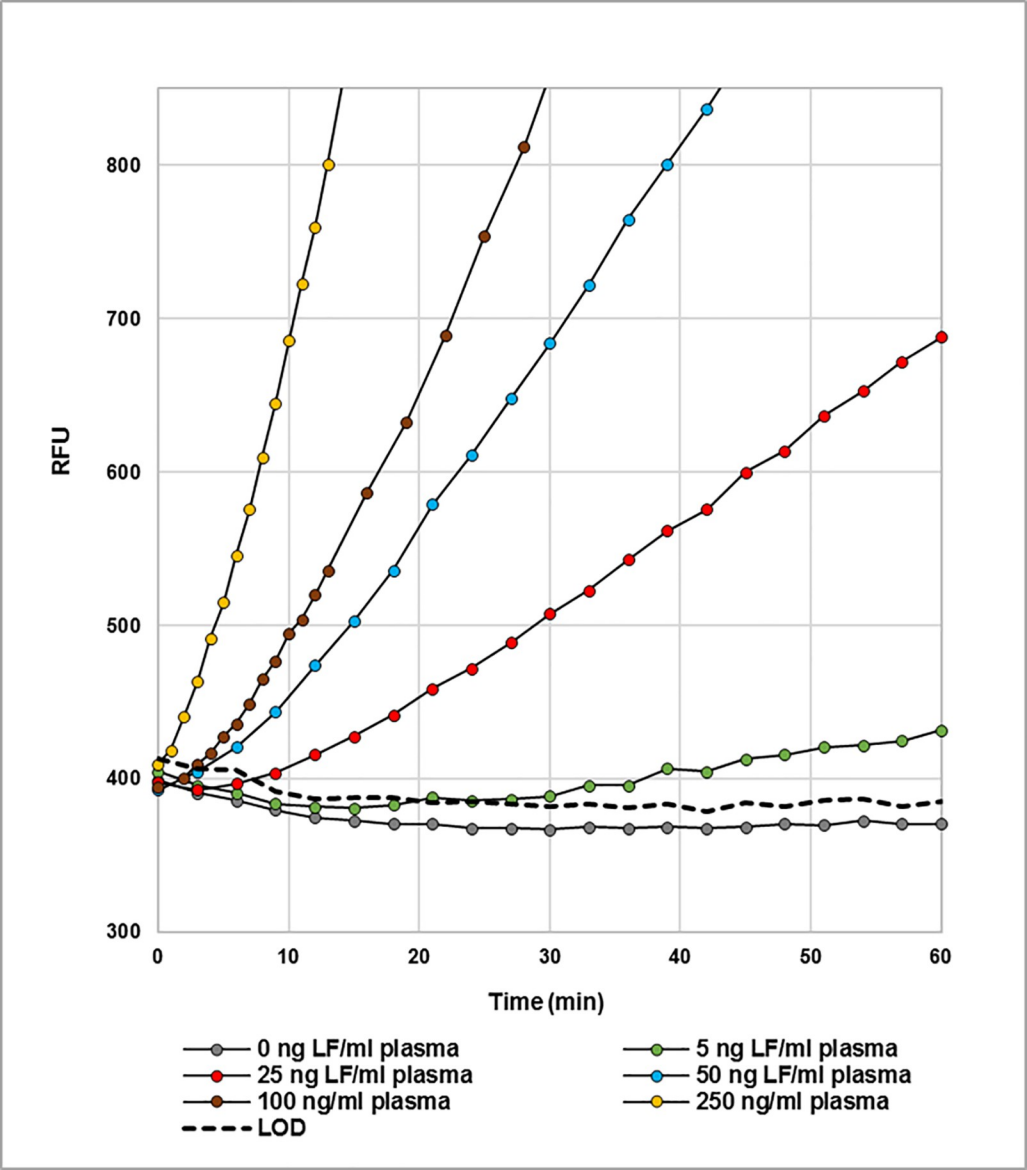
Cleavage of 10 μM MAPKKide Plus by a series of concentrations of LF(ng/ml) in bovine plasma as a function of time. The data shown are from the microplate reader using the kinetic mode.

Since significant levels of LF in plasma of infected individuals are detectable early on in the infection cycle [10, 14, 29], we also determined how rapidly high levels of LF could be detected using this simple microplate assay. **Fig 11** shows the increase in fluorescence with time by a series of LF concentrations in bovine plasma. Samples were prepared in plasma and diluted 1:5 with reaction buffer. Samples without LF in 1:5 diluted plasma are represented by the gray circles, and the LODs at each time point are shown by a black dashed line. After correction for dilution, samples with high levels of LF, 250 and 100 ng LF/ml plasma are detected in 2 and 5 minutes, respectively. Samples of LF in bovine plasma containing 50 ng LF/ml plasma are detected in 10 minutes while samples containing 25 and 5 ng/ml are detected in 15 and 45 minutes, respectively.

#### Microplate assay method using antibody capture in tubes to concentrate the LF and increase sensitivity

To improve the sensitivity of the microplate assay, capture of LF from undiluted plasma using antibody-coated immuno-tubes was investigated. Maxisorp immuno-tubes, with their greater surface area and volume, were used. In addition to enrichment of the LF, these tubes allowed capture of small amounts of LF from bovine plasma without dilution. After addition of the MAPKKide Plus, samples were monitored at 3, 5 and 22 hrs of incubation at 37°C using a fluorescent microplate reader.

**Fig 12:**
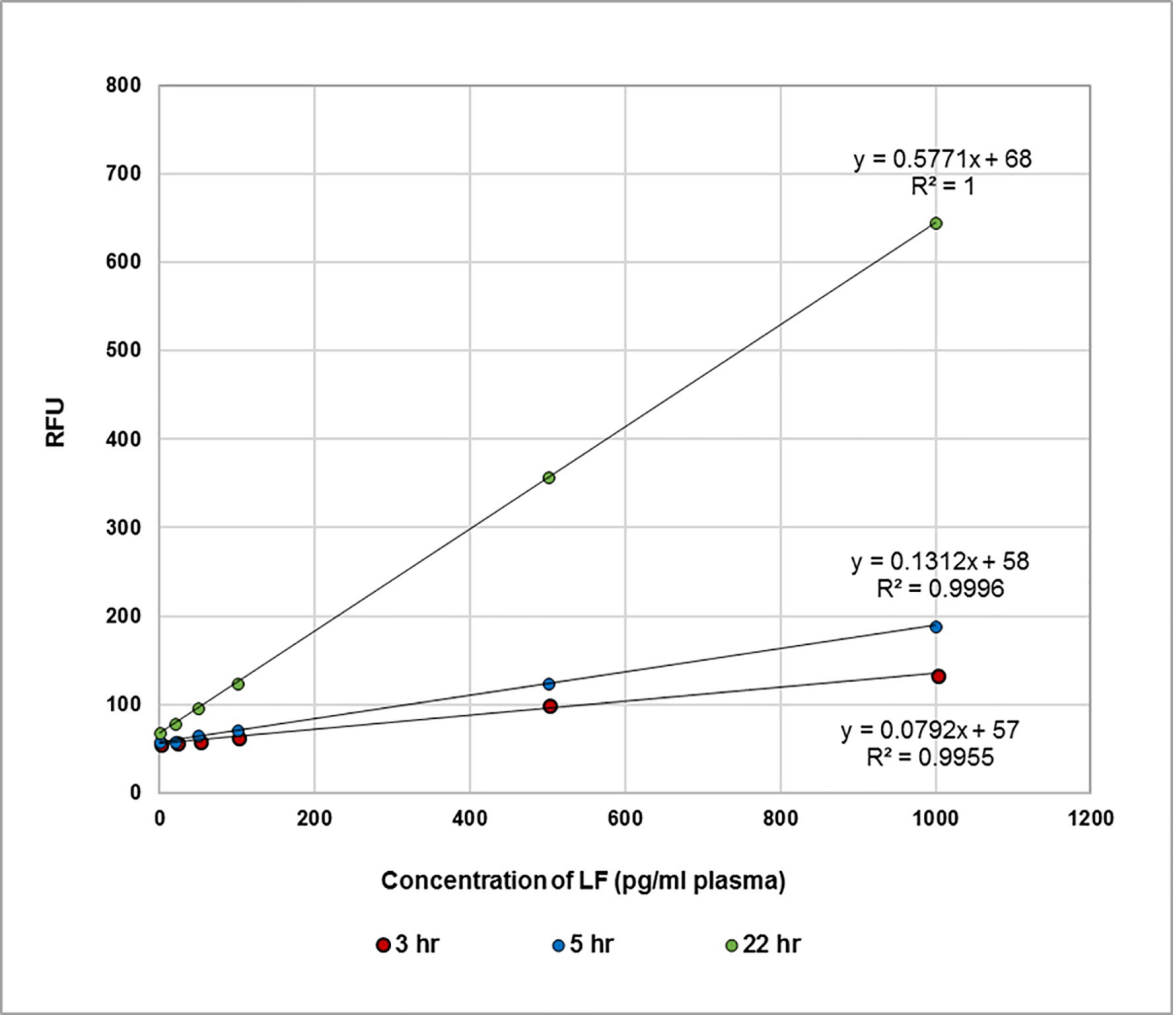
Cleavage of MAPKKide Plus monitored in the microplate assay. Plot of RFU versus concentration of LF (pg/ml bovine plasma) for 3 hour (green), 5 hour (blue), and 22 hour (red) digestion of MAPKKide Plus.

The data obtained for each time point (3, 5 and 22 hours) for 0 to 1000 pg LF/ml neat bovine plasma is given in **Table 7**. As shown in **Fig 12** the response is linear. The limit of detection after 5 hours is approximately 20 pg LF/ml of neat plasma.

**Table 7:** Fluorescence observed for the cleavage of MAPKKide Plus after 3, 5 and 22 hours exposure to LF captured from neat bovine plasma.

LF (pg/ml plasma)	RFU*			Slope (RFU/hour)
	3 hr digest	5 hr digest	22 hr digest	
0**	57	58	68	0.583
**20**	58	59	78	1.078
**50**	60	65	97	1.922
**100**	64	72	124	3.120
**500**	101	124	358	13.619
**1000**	134	189	645	26.867
**Slope RFU/(pg LF/ml)**	0.079	0.131	0.577	
**Intercept**	57	58	68	
**R**^**2**^	0.9955	0.9996	>0.9999	
Std deviation of the blanks**	0.69	0.69	2.46	
**Mean + 3 stdev**	58.6	60.3	75.6	
LOD (pg LF/ ml plasma)***	**21**	**18**	**13**	

*Samples are the average of three measurements.

**For the blanks there were three sets of triplicates and the standard deviation was calculated from these 3 sets of triplicates (n = 3).

***The limit of detection was calculated from the normal distribution of the blank samples (mean + 3 stdev; n = 3 triplicates) calculated as pg LF/ml neat plasma.

#### Kinetic analysis of the LF activity using the antibody capture microplate assay

**Fig 13:**
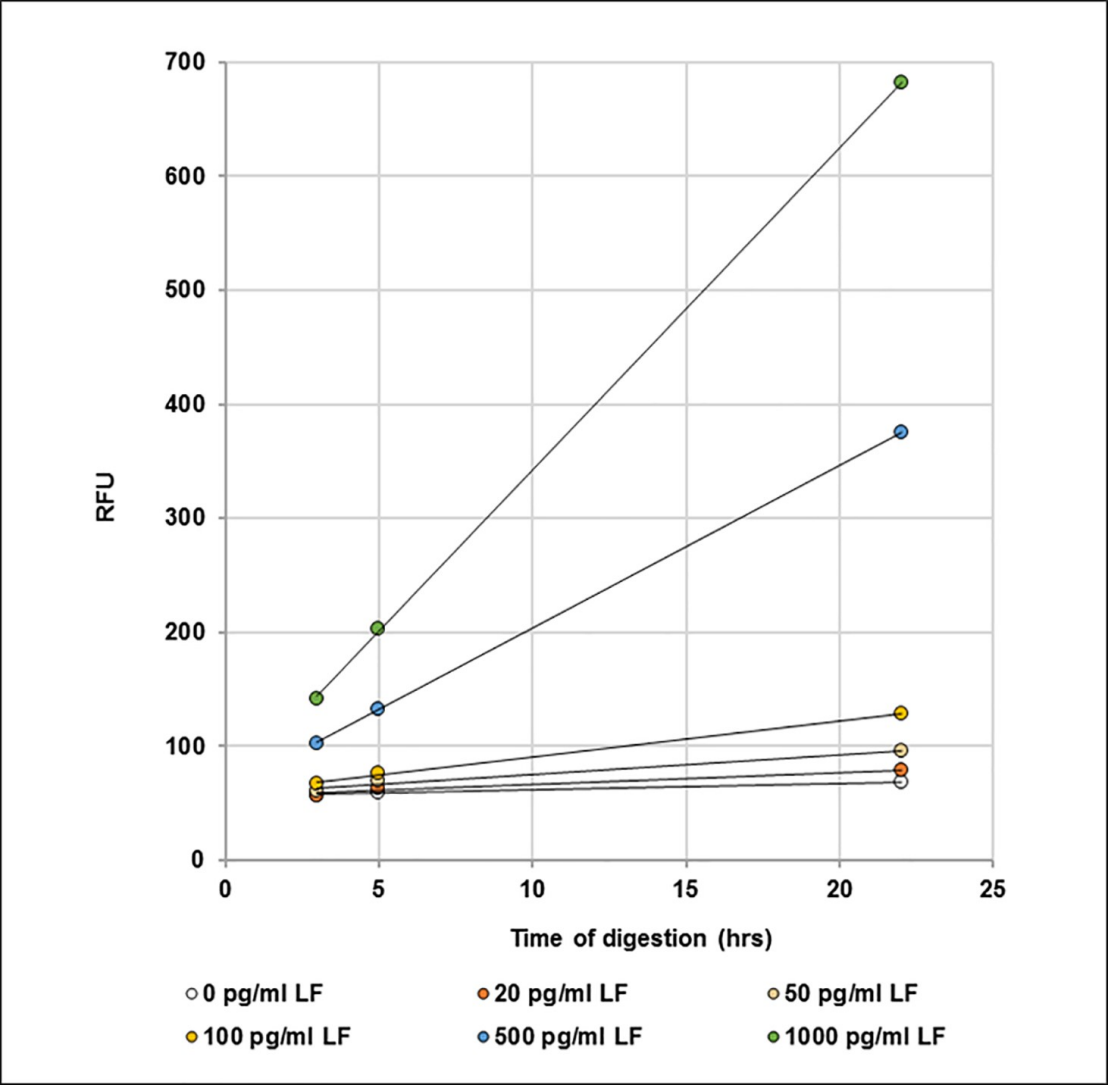
Kinetic analysis of microplate assay data. Cleavage of MAPKKide Plus by a series of concentrations of LF(pg/ml) in bovine plasma as a function of time.

**Fig 14:**
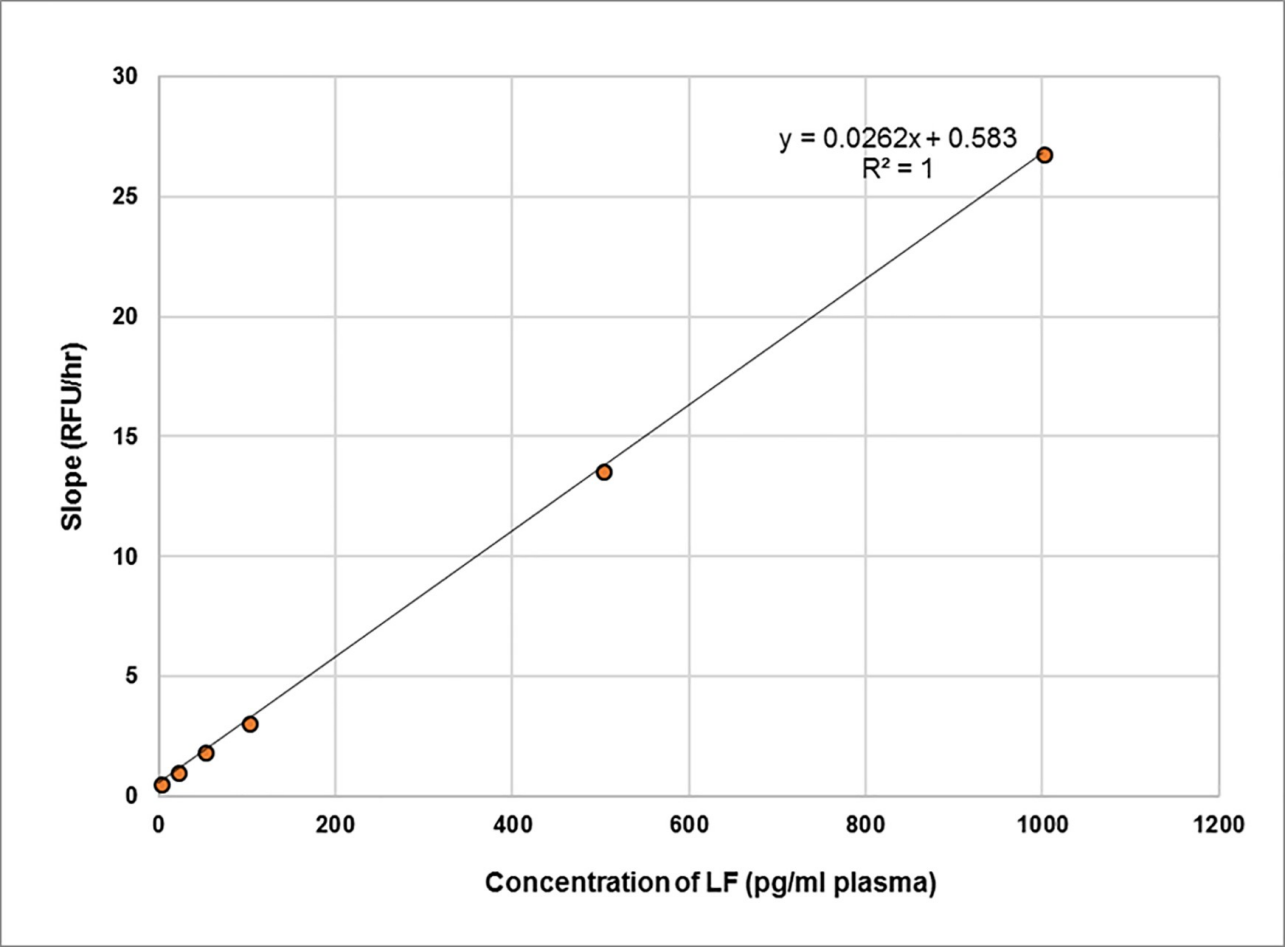
Rate of cleavage of MAPKKide Plus as a function of LF concentration. Slope in RFU/hour as a function of concentration of LF in neat bovine plasma.

The data above is for a single endpoint reading after 3, 5 or 22 hours. As discussed previously, these results can be confirmed by monitoring the rate of cleavage. **Fig 13** shows cleavage as a function of time for individual LF concentrations. The slopes, unique for each concentration, can then be plotted as a function of the concentration of LF and used to determine the amount of LF present in unknown samples (**Fig 14**). This accommodates differences in the background observed for different sources of plasma.

## Discussion

The results reported here describe the design of fluorogenic peptide substrates which are specific for LF in bovine plasma. Starting with the optimal 15 amino acid cleavage motifs identified by Turk et al. [17] and taking advantage of the fact that LF will allow a fluorophore at the site of cleavage [24], six potential short peptide substrates were designed and evaluated for specificity, efficiency of cleavage and minimum number of amino acids necessary. Since plasma is known to contain a number of proteases, the peptides were designed with D-amino acids at the sites presumed to be most vulnerable to non-specific cleavage, i.e. the two arginines and two lysines. As shown in **Fig 1**, the cleavage of two of these sequences, LBL 10097 and LBL 10081 (MAPKKide Plus), by LF in assay buffer is not affected by the presence of the D-amino acid substitutions when compared to the same sequence without D-amino acids, LBL 10079. The fluorescence signal originates from the release of the 7-amino-4-methylcoumarin from the C-terminal proline by LF. Cleavage at any other site does not result in increased fluorescence. However, as shown in **Fig 2**, while cleavage of each of the peptide substrates, LBL 10081 (MAPKKide Plus) and LBL 10097 in bovine plasma was dependent upon the presence and amount of LF, LBL 10079 was cleaved even in the absence of LF. These data suggest the presence of proline proteases in the plasma and also indicate that LBL 10081 (MAPKKide Plus) and LBL 10097 are resistant to such cleavage due to the use of D-amino acids on the N-terminus of these substrates. Interestingly, these peptides did not require the D analog of the proline in order to become resistant to non-specific proline protease cleavage. Also the results for LBL 10100 in **Figs 1** and **2**, indicate that a substrate as small as 7 amino acids retains activity.

These findings were followed by the evaluation of several methods for the detection of LF in plasma using the LBL 10081 (MAPKKide Plus) fluorogenic substrate. Two methods involving capture and enrichment of the LF from plasma are described, one HPLC-based and one a microplate assay. Using the HPLC method, LF levels as low as 5 pg/ml plasma are detected after 2 hours of incubation. The method, simplified to use a fluorescent microplate reader with capture of the LF from plasma using antibody coated immuno-tubes, can detect 20 pg LF/ml plasma after 5 hours of incubation. This study also describes a method where the substrate is added directly to 1:10 diluted plasma and cleavage monitored for an increase in fluorescence as a function of time using a fluorescent microplate reader. The limit of detection by this simple method, without the use of a capturing antibody and using a 1:10 dilution of the plasma, is <1 ng LF/ml neat plasma after 5 hours of digestion. This detection limit is well below the average level (40 ng/ml) found in one study of rhesus macques 48 hours post infection by inhalation [10] or another study where levels of LF ranging from 7 to over 100 ng/ml plasma were detected in rhesus macaques 36 hours after exposure [15]. Also, in a single case of inhalation anthrax in a human, over 200 ng/ml was detected 2–3 days after onset of symptoms [29]. In the current study, it was also shown that higher levels of LF, i.e. 250, 100, 50, 25 and 5 ng LF/ml plasma are detected in 2, 5, 10, 15 and 45 minutes, respectively, from a 1:5 dilution of plasma, direct addition of MAPKKide Plus, and kinetic readout from a microplate reader.

In conclusion, this report describes fast, sensitive, specific and accurate methods to detect active infection by *Bacillus anthracis* in plasma at very early stages of intoxication.
